# No evidence to support the use of glycerol–oxalic acid mixtures delivered via paper towel for controlling *Varroa destructor* (Mesostigmata: Varroidae) mites in the Southeast United States

**DOI:** 10.1093/jisesa/iead097

**Published:** 2023-12-06

**Authors:** Lewis J Bartlett, Christian Baker, Selina Bruckner, Keith S Delaplane, Ethan J Hackmeyer, Chama Phankaew, Geoffrey R Williams, Jennifer A Berry

**Affiliations:** Department of Entomology, University of Georgia, Athens, GA 30602, USA; Center for the Ecology of Infectious Diseases, Odum School of Ecology, University of Georgia, Athens, GA 30602, USA; Department of Entomology and Plant Pathology, Auburn University, Auburn, AL 36849, USA; Department of Entomology and Plant Pathology, Auburn University, Auburn, AL 36849, USA; Department of Entomology, University of Georgia, Athens, GA 30602, USA; Center for the Ecology of Infectious Diseases, Odum School of Ecology, University of Georgia, Athens, GA 30602, USA; Department of Entomology, Faculty of Agriculture, Kasetsart University, Chatuchuk, Bangkok 10900, Thailand; Department of Entomology and Plant Pathology, Auburn University, Auburn, AL 36849, USA; Department of Entomology, University of Georgia, Athens, GA 30602, USA

**Keywords:** honey bee, varroa, oxalic acid, practitioner, extension

## Abstract

A significant amount of researcher and practitioner effort has focused on developing new chemical controls for the parasitic *Varroa destructor* mite in beekeeping. One outcome of that has been the development and testing of “glycerol–oxalic acid” mixtures to place in colonies for extended periods of time, an off-label use of the otherwise legal miticide oxalic acid. The majority of circulated work on this approach was led by practitioners and published in nonacademic journals, highlighting a lack of effective partnership between practitioners and scientists and a possible failure of the extension mandate in beekeeping in the United States. Here, we summarize the practitioner-led studies we could locate and partner with a commercial beekeeper in the Southeast of the United States to test the “shop towel–oxalic acid–glycerol” delivery system developed by those practitioners. Our study, using 129 commercial colonies between honey flows in 2017 split into 4 treatment groups, showed no effectiveness in reducing *Varroa* parasitism in colonies exposed to oxalic acid–glycerol shop towels. We highlight the discrepancy between our results and those circulated by practitioners, at least for the Southeast, and the failure of extension to support practitioners engaged in research.

## Introduction

Despite their importance, honey bee populations in Europe and North America have been in decline or struggling to meet market demand, due in large part to management regimes, declining forage quality, agrochemical exposure, (re-)emerging parasites, and the insidious interaction of these various stressors (Smith et al. 2013, Goulson et al. 2015, Bruckner et al. 2023). In the United States, the major cause of western honey bee (*Apis mellifera* L.) population loss is the invasive *Varroa destructor* (Anderson and Trueman), an ectoparasitic mite that feeds on the fat body of honey bees and serves as a vector for certain viruses (Ramsey et al. 2019, Traynor et al. 2020, Wilfert et al. 2020). *Varroa* shifted its host from *A. cerana* to *A. mellifera* during the first half of the 20th century, resulting in an epidemic of re-emerging deformed wing virus (Wilfert et al. 2016). As one of the most prolific and harmful honey bee parasites, a large body of research has been invested in *V. destructor* control and continues to be a focus of beekeeping research (Bartlett 2022).

In the United States, there are currently 3 synthetic acaricides licensed for use in controlling *V. destructor* populations: amitraz, coumaphos, and (*tau*-)fluvalinate with others under research (Haber et al. 2019, Bahreini et al. 2020, Jack et al. 2021). However, *Varroa* have evolved resistance to all 3 available synthetic acaricides (Guo et al. 2021, Millán-Leiva et al. 2021, Vlogiannitis et al. 2021), with these treatment options exhibiting reduced effectiveness against mite populations (Rinkevich 2020). Alternative chemical treatment options include naturally derived acaricides, such as the widely used oxalic acid (ethanedioic acid). Oxalic acid is commonly applied through trickling, vaporization, or spraying (Rosenkranz et al. 2010, Jack and Ellis 2021). However, these acute application methods do not kill *V. destructor* that are located within cells of developing honey bee brood where they reproduce (Al Toufailia et al. 2015, Berry et al. 2022), as the cells are protected by waxy caps. These methods are often also time and labor intensive and can require specialized equipment, prohibiting widespread use (Bartlett 2022).

Due to these shortcomings in oxalic acid application techniques, new methods of extended-release application of oxalic acid are necessary to ensure the elimination of mites in both the brood and the hive at large (Maggi et al. 2016, Rodríguez Dehaibes et al. 2020). Successful development of glycerol-based oxalic acid solutions for *Varroa* control is documented (Maggi et al. 2016, Rodríguez Dehaibes et al. 2020, Sabahi et al. 2020, Kanelis et al. 2023). Paralleling this scientific research into organic acaricides for beekeeping, there has been increased interest in the experimental control of *Varroa* by practitioners or industry beekeeper communities. Publications popular among beekeepers have published multiple articles detailing practitioner-led inquiries into the effectiveness of different extended-release oxalic acid applications, which are read by scientists but often absent from our own reviews of the literature. One *Varroa* control method explored by the practitioner community is the use of a “shop towel” (a hard-wearing paper towel product) for oxalic acid extended-release. In this method, oxalic acid dissolved into glycerin over heat is poured onto a shop towel, which is then applied to the hive, often on the top bars of the lower brood box’s frames. The use of these home-made soaked shop towels is currently illegal in the United States. However, identifying the effectiveness of this method is important to open paths towards legal slow-release oxalic acid technologies. Sabahi et al. (2020) showed some success with this practitioner-developed method to control *Varroa* when tested at a small scale in Ontario, Canada.

Given practitioner interest in delivering oxalic acid via glycerol (Glycerol and glycerin(e) are both used in this article, they are interchangeable names for the same chemical and usually reflect context of purchase and use.) suspensions in colonies, we evaluated the possible effectiveness of this popular extended-release oxalic acid method in 2 ways. We undertook a large experimental trial following the practitioner-published methodology and using colonies provided by a commercial beekeeper in South Georgia, USA. We searched for all instances of practitioner-led studies on this technique, with a mind to undertaking a reanalysis of those results in composite; however, we were unable to achieve this latter approach satisfactorily.

## Methods

### Field Experiment

We worked with a commercial beekeeper in South Georgia between honey flows to test extended-release oxalic acid treatments during the summer foraging dearth under realistic field conditions. We identified queenright, otherwise healthy colonies from 12 to 14 June 2017. Colonies were included for analysis if they remained queenright and alive throughout the 4-week experiment, with 129 colonies total across the analysis. To assess mite parasitism levels at the beginning of the experiment, adult bees from brood frames were shaken into collection trays so that approximately 300 bees could be collected into 70% ethanol solution for *Varroa* assessment. Colonies within yards were, at this time, assigned to one of 4 treatment groups: negative control (CTRL) with no intervention, high oxalic acid (HOA), medium oxalic acid (MOA), and sham (0-OA) receiving shop towels containing 18, 12, and 0 g of oxalic acid, respectively. The 129 colonies were divided unevenly between 4 apiaries and 4 treatments (all treatments present in all apiaries); 38 colonies received the “HOA” treatment, 39 received the “MOA” treatment, 28 received the “0-OA” treatment, and 24 colonies received the “CTRL” treatment. Larger sample sizes were biased towards groups receiving oxalic acid to reduce the economic impact on the collaborating commercial beekeeper as best as possible. At the start of the experiment, mean parasitism rates (mites per 100 bees or ‘percent mite intensity’, PMI) across the 129 colonies were 2.1, and the median was 1.15. There was no significant difference in starting PMI values between assigned treatment groups (ANOVA: *F*_3,125_ = 0.25; *P* = 0.0.863). Apiaries (yards) were separated by multiple miles from one another, and colonies within apiaries were separated by a few feet typical of a normal commercial apiary.

Oxalic acid-impregnated shop towels were made 1 day prior to application using a modified protocol based on studies in Supplementary Table S1 and Maggi et al. (2016). In brief, 715-ml distilled water (Nice!, Deerfield, IL) was slowly brought to 83 °C, then added to a beaker containing 660 g oxalic acid dihydrate (Brushy Mountain Bee Farm, Moravian Falls, NC), which was heated using a hot plate (VWR, Radnor, PA) set to 325 °C until the oxalic acid dihydrate was fully dissolved. Temperatures were monitored using a calibrated thermometer (VWR, Radnor, PA). Next, 550-ml vegetable glycerin (Froggy’s Fog, Columbia, TN), previously warmed in a microwave (Sunbeam, Boca Raton, FL) for 1 min, was added, and the solution was homogenized by stirring. The solution was allowed to cool, then poured onto a 13.95 × 13.2 cm 55-sheet-roll of blue shop towels (Scott, Neenah, WI) that was previously cut in half transversely and placed in a metal cake pan (Mainstays, Bentonville, AR). The fully soaked towel roll, containing 55 sheets cut in half, was left in a ventilated room overnight to allow the evaporation of excess moisture. Sham shop towels (0-OA treatment) were made using the same protocol but omitting the oxalic acid.

Shop towel sections were deployed in colonies by placing them on the top bars of the lower brood box’s frames, with the upper brood box placed on top, and were left in the colony for 42 days. Colonies were dosed with either 0, 12, or 18 g of oxalic acid, where 0-g colonies received 3 shop towel sections containing only glycerol, while 12- and 18-g colonies received 2 or 3 shop towel sections with the glycerol–oxalic acid mixture, respectively (6 g per shop towel section). Mite parasitism levels were measured again at the end of the treatment period for each colony.

### Statistical Analyses

All data handling and analysis were undertaken in R (R Core Team 2019) v.3.6.1. We provide a Zenodo-archived GitHub repository with all analysis and data freely available (DOI: 10.5281/zenodo.8381423). For each colony, we calculated the change in percent mite intensity between the start and end of the treatment period (ΔPMI). We analyzed this ΔPMI response variable using linear mixed-effects modeling and type-III ANOVA approach, following the “afex” package (Singmann et al. 2019), which wraps around the “lme4” (Bates et al. 2015) package. In all cases, we included “Yard” (apiary) as a random effect and a single fixed predictor, with ΔPMI as the response variable. In the first instance, we analyzed the full dataset with “treatment” as an unordered factor as the predictor. We followed this with analyses of data subsets, in one case using the binary predictor of whether a glycerin towel was present (True/False) on only the CTRL and 0OA treatments, and in a second instance, we used a continuous “dose” predictor of either 0, 12, or 18 g of oxalic acid on a data subset including the 0OA, MOA, and HOA treatments.

### Narrative Review and Reanalysis

We comprehensively reviewed US-based practitioner articles from 2015 onwards for data on slow-release oxalic acid for *Varroa* control, specifically the “shop-towel” method or similar approaches using other cellulose matrices (e.g., cardboard strips), to identify the breadth of testing undertaken by practitioners using this technique. We initially anticipated undertaking a meta-analysis of this literature; however, we withdrew that approach on the basis of the few number of studies, all by one author, and the availability of the required data to undertake a reanalysis.

## Results

### Experimental Trial

**Fig. 1.**
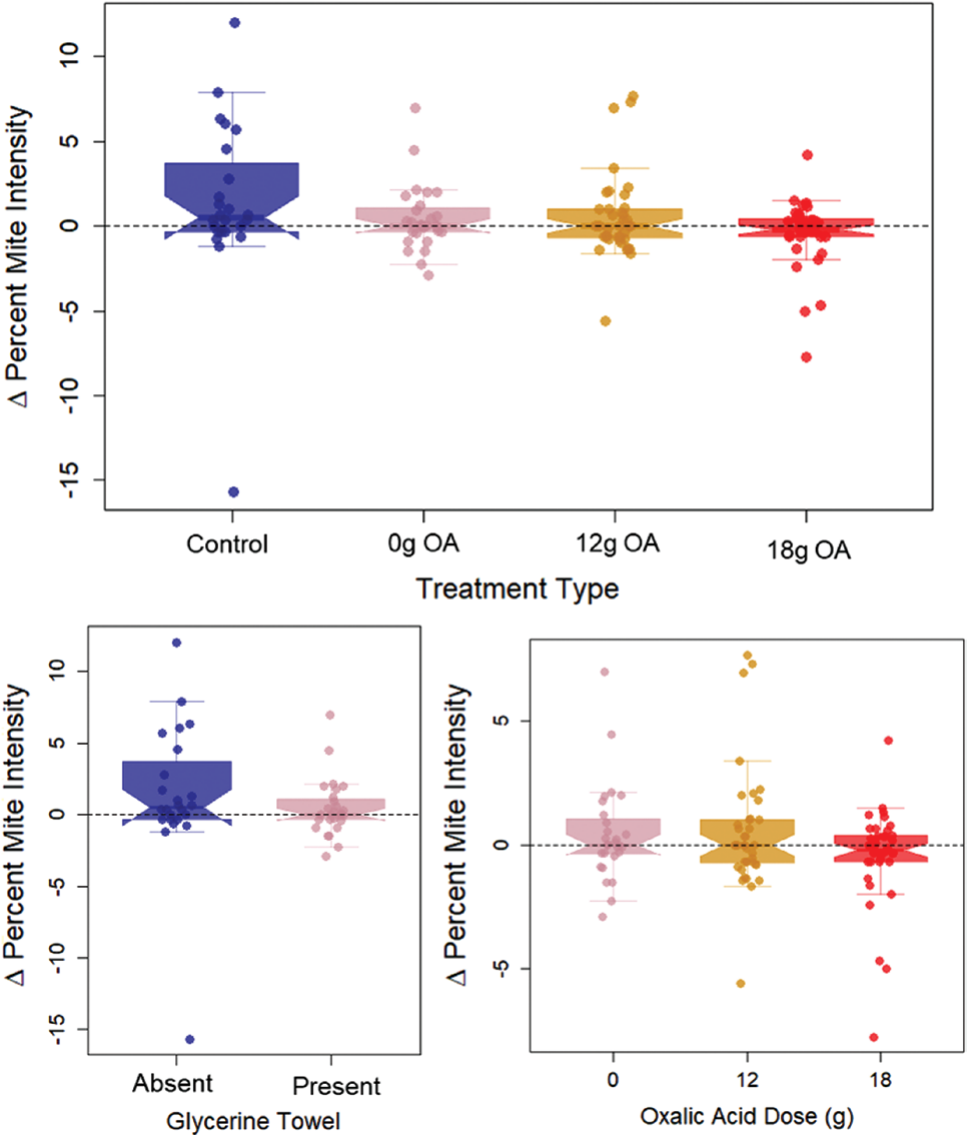
∆PMI for all colonies (points) across treatment groups (colored box plots). (a) The full data set and analysis of each treatment as its own unordered factor. (b and c) Data subsets of (a), reflecting alternative analyses. In (b), we compare the effect of the glycerin towel being present (CTRL vs. 0 g OA), and in (c), we analyzed only colonies with glycerin towels based on oxalic acid dose (0 g OA vs. 12 g OA vs. 18 g OA). Coloring of treatment groups is consistent across panel.

All experimental results are presented in Fig. 1. Across the full dataset, we found no clear evidence of differences between treatment groups in their change in per-capita mite parasitism (delta percent mite intensity, ∆PMI), when using a linear mixed-effects model and corresponding ANOVA, with “∆PMI” as the response variable, “Treatment (factor)” as the only fixed predictor, and “Yard” as random effect (*F*_3,121.6_ = 1.76; *P* = 0.158). Correspondingly, we found no evidence of glycerin-oxalic acid shop towels affecting ∆PMI when we analyzed a data subset including only colonies with towels (HOA, MOA, and 0-OA), where we used the same modeling approach as above, however used a “Dose (continuous)” fixed effect instead of treatment (*F*_1,102.1_ = 2.32; *P* = 0.131). We further found no effect on ∆PMI from exposure to the glycerin towel, comparing the “no intervention” negative control (CTRL) to the 0-OA treatment, again as above using the corresponding data subset and a binary “GlycerineTowel” fixed predictor variable (*F*_1,43.83_ = 0.76; *P* = 0.389).

Across the whole experiment, ∆PMI remained on average close to 0. Only the “CTRL” group (no intervention) showed on average a marginal increase in ∆PMI of +1.34 (0.19 to 2.49 95% confidence interval [CI]). The remaining groups (0-, 12-, and 18-g oxalic acid in glycerin towels) showed +0.41 (−0.65 to 1.47 95% CI), +0.50 (−0.40 to 1.40 95% CI), and −0.43 (−1.34 to 0.48 95% CI) average ∆PMI values, respectively. Quoted numbers are linear mixed-model coefficients and corresponding 95% CI ranges.

### Narrative Review and Reanalysis

We identified 8 published studies in the practitioner publication “American Bee Journal” spanning 2017–2021, all by R. Oliver. *American Bee Journal* is a wide-reaching practitioner journal and arguably comes under the “gray literature” umbrella. Differences in approach to publishing studies between the beekeeper literature and the academic literature, including around data ownership, study design, response variables, and presentation of results, prevented reanalysis.

## Discussion

We found no significant evidence that extended-release oxalic acid shop towels reduced mite levels at either treatment dose. We found mixed evidence overall that mite populations increased over the duration of the experiment. Though we found that the negative control group’s ∆PMI was higher than 0, none of the treatments were significantly different from one another. We could not present a reanalysis of practitioner-published data, as no described studies met the criteria of adequate reporting and correct experimental design.

Data from our experiment does not support the use of a “home-made” shop towel delivery for extended-release oxalic acid application in hives, at least in the Southeast United States and at the doses used here. This is in contrast to the results of Sabahi et al. (2020) in Canada. It is plausible that the climate, notably the high humidity, in the Southeast interferes with this mite control method (Patricia et al. 2013, Gregorc et al. 2017). The sites in our test region during the month of the experiment saw daily average highs of 34 °C and average lows of 22 °C, with an average relative humidity of 76% across the day, typical for the time of year. Any further direct comparisons or speculations are difficult, as Sabahi et al. (2020) only tested 10 colonies when considering an oxalic acid—glycerin mixture (5 control, 5 treated). Additionally, our results contrast with beekeeper-led experiments in California (Supplementary Table S1); however, the response variables and experimental designs significantly differed from ours, which may not be the cause of the discrepancies but does limit our ability to draw insightful comparisons. However, the same climate variables may be a leading cause of our failure to recreate the documented results. Similar if differently formulated products, such as Aluen CAP, studied by Maggi et al. (2016) in Argentina, may or may not face the same discrepancies in our region and may not suffer from the same discrepancies; studies on those products have recently been completed for this region (Aurell D et al., personal communication).

The discrepancies between our scientific study and the practitioner-led ones could indicate that cooperative extension agents fail to adequately support practitioners who wish to contribute and experimentally test novel control applications. Without guidance, experiments can be limited by the ability of regulators or research colleagues to make use of the results (Supplementary Table S1). We consider this a failing of the extension mission, where better relationships between scientists and practitioners could have led to effective collaboration, making the best use of the considerable resources practitioners are willing to devote to science. This is a gross inefficiency on our part as industry-serving scientists, not only concerning wasted colonies and details of experimental design, but also lost time, effort, and personnel needed to communicate contradictory results or explain nuance once adequate field testing has been completed. By better supporting those beekeepers willing to donate time and resources, extension and research programs can ensure that community science remains robust and widely implemented. The resources devoted to scientific enquiry by Oliver and other industry/stakeholder practitioners, including the commercial beekeeper who collaborated in this study, are significant and show that the industry pursues the same goals as the applied academic research-funding-extension engine. The disconnect between scientists and stakeholders has been previously identified in the beekeeping/bee science sector. The international ‘COLOSS’ bee research program has highlighted their need to break down the “Ivory Tower,” with thoughtful from Fabricius Kristiansen et al. (2022) explaining the need to achieve the ‘win-win’ situations sought in research-practitioner partnership. We note that this study was conducted using commercial apiaries, in collaboration with willing beekeepers, in the spirit of that message.

Our results support an ongoing effort to establish “off the shelf ready” formulations of oxalic acid to help control *Varroa* (Rodríguez Dehaibes et al. 2020, Kanelis et al. 2023), and caution against the use, adoption, or recommendation of “home-brew” *Varroa* control techniques that are off-label and published in nonacademic sources. We also highlight that without better bridges between researchers and beekeepers, efforts will continue to be wasted. Scientists should consider prioritizing how to capitalize on the willingness of beekeepers to run scientific studies when developing new mite control methods or formulations.

## Supplementary Material

iead097_suppl_Supplementary_Tables_S1

## Data Availability

All data and analysis scripts will be made available as a Zenodo-deposited GitHub repo, and cited appropriately, upon manuscript finalization and acceptance.
