# Supplementary material for: No evidence to support the use of glycerol–oxalic acid mixtures delivered via paper towel for controlling Varroa destructor (Mesostigmata: Varroidae) mites in the Southeast United States
Source: J Insect Sci. 2023 Dec 6;23(6):18. doi: 10.1093/jisesa/iead097 (PMC10699872; doi:10.1093/jisesa/iead097)
Supplement: iead097_suppl_Supplementary_Tables_S1 [file iead097_suppl_supplementary_tables_s1.docx]

**Supplementary Information**

**No evidence to support the use of glycerol - oxalic acid mixtures delivered via paper towel for controlling *Varroa destructor* (Mesostigmata: Varroidae) mites in the Southeast United States.**

Lewis J. Bartlett^1,2^*, Christian Baker^3^, Selina Bruckner^3^, Keith S. Delaplane^1^, Ethan J. Hackmeyer^2^, Chama Phankaew^4^, Geoffrey R. Williams^3^, Jennifer A. Berry^1^

1 Department of Entomology, University of Georgia, Athens, GA 30602 USA
2 Center for the Ecology of Infectious Diseases, Odum School of Ecology, University of Georgia, Athens, GA 30602 USA
3 Department of Entomology & Plant Pathology, Auburn University, Auburn, AL 36849 USA

4 Department of Entomology, Faculty of Agriculture, Kasetsart University, Chatuchuk, Bangkok 10900, Thailand

| **Table S1** - Summary of slow-release oxalic acid practitioner studies (all published without in American Bee Journal (ABJ) by Oliver based in California), representing a large investment of time and colonies, which guided our experiment in Georgia (US). | | | | | | |
| --- | --- | --- | --- | --- | --- | --- |
| ***Identified Study*** | ***Publication Location & Date*** | ***Compares Control & Treated*** | ***Colony Assignments*** | ***Response Variable*** | ***Sample Sizes?*** | ***Measures of Spread?*** |
| Beyond Taktik | ABJ, January 2017 | Yes | High estimated mite loads assigned to treated group | Mite counts | Yes | No |
| Extended Oxalic Acid Progress Report #2 | ABJ, October 2017 | No | No Control | % change in mites | Control - No, Treated - Yes | No |
| Extended Oxalic Acid Progress Report 2018, California Field Trial | ABJ, November 2018 | Yes | Unclear | % change in mites | Yes | No |
| Extended Oxalic Acid Progress Report #3 | ABJ, January 2018 | Yes | High estimated mite loads assigned to treated group | Mite counts | Yes | No |
| Extended Oxalic Acid Progress Report #4 | ABJ, November 2018 | Yes | High estimated mite loads assigned to treated group | Mite counts (for subset) | Yes | Yes |
| Extended Oxalic Acid Progress Report #5 | ABJ, December 2019 | No | No Control | Mite counts | Yes | No |
| Mite Control While Honey is Still on the Hive Part 2 | ABJ, December 2020 | Yes | Unclear | Mite counts | No | No |
| Mite Control While Honey is Still on the Hive Part 4 | ABJ, February 2021 | No | No Control | Mite drop | Yes | No |
